# “Medical Notice!

**Published:** 1849-07

**Authors:** Saml. Boicourt


"MEDICAL NOTICE!

The undersigned begs leave to inform the whole world, that he is now qualified to ameliorate the wretchedness, distress, and painful affliction of which the whole human family are more or less suffering. It has been, and now is, and hereafter can be proved, that my skill in *Dental Surgery*, and universal cures of all diseases, is unfathomable and infinite.

For proof, I appeal and refer to the most learned, scientific, and philosophic of the medical faculty, from Mount Aristook, north, to the Sabine, south; and Nova Scotia, east, to Nootka Sound, west, indiscriminately. It is not merely bragging to say that I can extract teeth without pain, the most decayed, and largest, and more of them from one mouth than any other man under heavens; and can replace new and sound ones, of eternal durability and youthful appearance, in the least time. Toothless old maids can be armed with retaliating weapons to bite silly and neglectful old bachelors, and bring them to a sense of their duty, and make 'em fork over.

My cathartic medicinal pills, are so certain, speedy, and effectual, that the most public gentleman will be in want of a private room, to which a rail-road and locomotive are indispensably necessary to facilitate his speedy retreat, and return in prime health to his friends, rejoicing.

SAML. BOICOURT.

☞ Office on Fifth street, No. 6, west side, between Main and the Ohio river, Louisville, Ky."

NOTE.—The above sent us by a friend, we insert, although an advertisement, gratis. If the gentleman wishes a further continuance, we shall charge according to rule.—CIN. ED.

